# Prevalence and Pattern of Hemoglobinopathies Among Women of Reproductive Age Attending a Tertiary Care Center in Central Uttar Pradesh: A Prospective Observational Study

**DOI:** 10.7759/cureus.116623

**Published:** 2026-09-21

**Authors:** Hera Khan, Neena Gupta, Renu Gupta, Pratima Verma, Preeti Tyagi, Garima Gupta, Lubna Khan

**Affiliations:** 1 Obstetrics and Gynecology, Ganesh Shankar Vidyarthi Memorial Medical College, Kanpur, IND; 2 Pathology, Ganesh Shankar Vidyarthi Memorial Medical College, Kanpur, IND

**Keywords:** hbe trait, hemoglobinopathies, high-performance liquid chromatography, women of reproductive age, β-thalassemia trait

## Abstract

Background

Hemoglobinopathies are an important public health concern in India, with considerable variation across regions. Screening women of reproductive age can help identify carriers early and provide opportunities for reproductive counselling. This study assessed the prevalence and pattern of hemoglobinopathies among women attending a tertiary care center in Central Uttar Pradesh.

Methods

A prospective observational study was conducted among women aged 15-49 years attending antenatal and outpatient services at a tertiary care hospital in Kanpur from 3 February 2025 to 2 February 2026. A total of 5,285 participants were screened. Those with abnormal or suggestive findings underwent further evaluation using complete blood count parameters and high-performance liquid chromatography. Diagnostic categories and hematological parameters were compared using appropriate statistical tests.

Results

Hemoglobinopathy was identified in 206 of 5,285 participants, giving an overall prevalence of 3.89%. Among the 207 participants with sufficient diagnostic information, beta-thalassemia trait was the most common finding (185; 89.4%), followed by hemoglobin E trait (20; 9.7%) and delta-beta-thalassemia trait (1; 0.5%). The prevalence of beta-thalassemia trait among the screened population was 3.5%. Significant differences were observed in red cell distribution width, hemoglobin, hemoglobin A, hemoglobin A2, and fetal hemoglobin (all p<0.001), while mean corpuscular volume and mean corpuscular hemoglobin did not differ significantly. Anemia severity also differed significantly (p=0.017).

Conclusion

Hemoglobinopathies were identified in a notable proportion of women screened, with beta-thalassemia trait being the predominant finding. These findings highlight the importance of carrier screening among women of reproductive age. The hospital-based design should be considered when interpreting the prevalence estimates.

## Introduction

Hemoglobinopathies are disorders of hemoglobin structure or synthesis and represent an important public health problem in the Indian population, with β-thalassemia being one of the most frequent disorders and showing significant interpopulation variation in carrier frequency [1,2]. A multicenter study conducted among 56,780 participants in India demonstrated the geographic distribution of β-thalassemia trait across six urban centers and different ethnic groups, while the prevalence of other hemoglobin variants also varied geographically [2]. Laboratory-based screening studies have also reported a wide range of hemoglobin variants identified by high-performance liquid chromatography (HPLC) in Indian populations [3].

Pregnancy screening provides an opportunity to identify carriers, followed by the assessment of partners and at-risk couples. In a seven-year antenatal screening study in India, carrier women were identified, their spouses were tested, and prenatal diagnosis was offered to couples identified as being at risk [4]. More recently, a prenatal screening program in India incorporated HPLC-based screening, partner testing, molecular confirmation, and prenatal testing for at-risk couples [5].

Hematological indices can help identify individuals who require further evaluation; however, iron deficiency may interfere with the interpretation of hemoglobin A2 (HbA₂) levels and complicate the laboratory evaluation of suspected β-thalassemia trait [3]. HPLC enables the detection and characterization of thalassemia and other hemoglobin variants through the analysis of hemoglobin fractions and retention times [3]. In a recent multicenter study, HPLC-based testing identified hemoglobinopathy carriers who would not have been detected when screening was initiated with complete blood count (CBC) [6].

Thus, the present study was conducted to determine the prevalence and pattern of hemoglobinopathies among women aged 15-49 years attending a tertiary care hospital in Central Uttar Pradesh, India, using hematological parameters and HPLC-based hemoglobin analysis.

## Materials and methods

Study design and setting

A prospective observational study was conducted to determine the prevalence and distribution of hemoglobinopathy traits among women of reproductive age attending the Department of Obstetrics and Gynecology at Upper India Sugar Exchange Maternity Hospital (UISEMH), Ganesh Shankar Vidyarthi Memorial (GSVM) Medical College, Kanpur, India, from 3 February 2025 to 2 February 2026. The hospital is a tertiary care center providing antenatal, intrapartum, and postnatal services.

Study participants

The study enrolled women aged 15-49 years attending antenatal and outpatient services during the study period. Written informed consent was obtained from all participants. Women outside the specified age range, those who refused consent, and those without adequate laboratory data for diagnosis were excluded.

A total of 5,285 participants were screened for hemoglobinopathy. All enrolled participants underwent hemoglobinopathy screening using HPLC. Participants with abnormal HPLC findings underwent further hematological assessment for diagnostic correlation and classification.

Process of data collection and hematological assessment

Demographic and clinical data, including age, religion, caste, socioeconomic status, consanguinity, and family history of hemoglobinopathy, were collected using a structured data collection format. Approximately 3 mL of venous blood was collected under aseptic conditions in ethylenediaminetetraacetic acid (EDTA) vacutainers.

CBC parameters assessed included hemoglobin, red blood cell count, mean corpuscular volume (MCV), mean corpuscular hemoglobin (MCH), mean corpuscular hemoglobin concentration (MCHC), and red cell distribution width (RDW). These hematological indices were interpreted along with the HPLC findings for hemoglobinopathy classification [7].

HPLC and diagnostic classification

HPLC was used for hemoglobin analysis with a Bio-Rad D-10 analyzer (Hercules, California, United States). Retention time, peak shape, and the percentages of hemoglobin A (HbA), HbA₂, fetal hemoglobin (HbF), and hemoglobin variants were evaluated from the chromatograms. HPLC is an established technique for the characterization of thalassemia syndromes and hemoglobin variants [7,8].

The diagnosis of β-thalassemia trait was based on the typical HPLC pattern, elevated HbA₂ levels, and corresponding hematological findings. An HbA₂ level >3.5% is typically suggestive of β-thalassemia heterozygosity [8]. HPLC patterns and hemoglobin fractions were used to classify HbE trait and other hemoglobin variants [7,8]. Participants with elevated HbF levels and an HPLC pattern suggestive of δβ-thalassemia were classified as having presumptive δβ-thalassemia trait based on HPLC findings and hematological correlation; molecular confirmation was not performed.

The diagnostic categories included β-thalassemia trait, HbE trait, and presumptive δβ-thalassemia trait based on HPLC findings and hematological correlation. Participants without an identified hemoglobinopathy were classified as having no identified hemoglobinopathy; this group included the participant diagnosed with iron deficiency anemia without hemoglobinopathy.

Assessment of anemia

The presence and severity of anemia were determined based on hemoglobin concentration. Anemia was classified as mild, moderate, or severe according to the hemoglobin cutoffs followed in the study: 10-10.9 g/dL for mild anemia, 7-9.9 g/dL for moderate anemia, and <7 g/dL for severe anemia.

Study outcomes

The primary outcome was the prevalence of hemoglobinopathy, calculated using the number of participants with identified hemoglobinopathy as the numerator and the total screened population (n=5,285) as the denominator. Secondary outcomes included the distribution of different hemoglobinopathy types among the diagnostic-analysis population, the severity of anemia according to diagnostic category, and differences in hematological and HPLC parameters between the diagnostic categories.

Quality control and bias reduction

Participant enrollment, data and sample collection, hematological assessment, HPLC testing, and diagnostic interpretation were performed using standardized procedures. Results were interpreted according to predefined hematological and chromatographic criteria. Invalid chromatographic runs were repeated, and the collected data were checked for completeness and consistency before analysis.

Sample size and missing data

No formal sample size calculation was performed before the study, as all eligible participants who underwent hemoglobinopathy screening during the study period were considered for inclusion. A total of 5,285 participants were screened, of whom 207 were included in the diagnostic analysis based on the availability of sufficient diagnostic information. No imputation was performed for missing data. Participants with missing information required for diagnostic classification were excluded from the corresponding analysis.

Statistical analysis

IBM SPSS Statistics for Windows, Version 27.0 (IBM Corp., Armonk, New York, United States) was used for data entry, coding, cleaning, and analysis. Categorical variables were presented as frequencies and percentages, while continuous variables were presented as mean±standard deviation.

The overall prevalence of hemoglobinopathy was calculated by dividing the number of participants identified as having a hemoglobinopathy by the total number screened (n=5,285). Individual diagnostic categories were described among the 207 participants included in the diagnostic analysis.

Associations between categorical variables and diagnostic categories were assessed using the chi-squared test or Fisher's exact test, as appropriate. One-way analysis of variance (ANOVA) was used to compare continuous hematological and HPLC parameters across the diagnostic categories. A two-sided p-value of <0.05 was considered statistically significant.

Ethical considerations

Ethical approval was obtained from the Institutional Ethics Committee for Biomedical Health and Research of GSVM Medical College, Kanpur, before the commencement of the study (approval number: EC/20/Feb./2025; approval date: 2 February 2025). Written informed consent was obtained from all participants before enrollment. Participant confidentiality was maintained throughout the study, and the collected information was used solely for research purposes.

## Results

According to Table 1, the study participants were largely distributed in the age category of 21-30 years with the age category of 26-30 years having the highest number of participants. The age group of 21-25 years had the second highest number of participants, while the age groups of ≤20 years and 31-35 years had comparatively fewer numbers of participants. Participants aged >35 years represented the least proportion of the study population.

**Table 1:** Age distribution of the study participants included in the diagnostic analysis

Maternal age group	Frequency (n=207)	Percentage (%)
≤20 years	19	9.1
21-25 years	71	34.3
26-30 years	78	37.7
31-35 years	30	14.5
>35 years	9	4.3

Among the 5,285 participants screened, 207 participants were included in the diagnostic analysis. Of these, 206 participants were identified as having a hemoglobinopathy, corresponding to an overall hemoglobinopathy prevalence of 3.89% (206/5,285). Among the diagnostic analysis participants, β-thalassemia trait was the most frequently identified condition (185/207, 89.4%), followed by HbE trait (20/207, 9.7%) and δβ-thalassemia trait (1/207, 0.5%). Additionally, one participant was diagnosed with iron deficiency anemia without evidence of a hemoglobinopathy (1/207, 0.5%). Thus, the final diagnostic analysis included 207 participants, comprising 206 individuals with hemoglobinopathies and one individual with iron deficiency anemia (Table 2).

**Table 2:** Overall prevalence and distribution of hemoglobinopathy traits HbE: hemoglobin E; IDA: iron deficiency anemia; β-thalassemia: beta-thalassemia; δβ-thalassemia: delta-beta-thalassemia

Diagnostic category	Frequency (n)	Percentage among diagnostic analysis participants (%)
Total screened cases	5,285	100
Hemoglobinopathy-positive cases	206	3.89
Sub-distribution of positive cases		
β-Thalassemia trait	185	89.4
HbE trait	20	9.7
Other variants (δβ-thalassemia trait)	1	0.5
No identified hemoglobinopathy		
IDA only	1	0.5

Comparisons were made regarding sociodemographic factors among the diagnostic groups. No significant relation was found between the diagnostic groups and consanguinity (p=0.131), religion (p=0.445), caste (p=0.320), or socioeconomic status (p=0.204). Participants in all these groups were mainly non-consanguineous and Hindus and had a lower-middle socioeconomic status. No participant had a family history of hemoglobinopathy (Table 3).

**Table 3:** Sociodemographic characteristics and their association with diagnostic category HbE: hemoglobin E; IDA: iron deficiency anemia; SC: scheduled caste; NA: not applicable; β-thalassemia: beta-thalassemia; δβ-thalassemia: delta-beta-thalassemia

Variable	Category	β-Thalassemia trait	HbE trait	No identified hemoglobinopathy (IDA only)	δβ-Thalassemia trait	Total (n)	P-value (Fisher's exact test)
Consanguinity	No	170	18	1	0	189	0.131
	Yes	15	2	0	1	18	
Religion	Hindu	149	16	1	0	166	0.445
	Muslim	36	4	0	1	41	
Caste	Thakur	19	4	0	0	23	0.320
	Brahmin	21	0	0	0	21	
	SC	12	1	0	0	13	
	Yadav	9	1	1	0	11	
	Ansari	10	1	0	0	11	
	Verma	10	1	0	0	11	
	Pathan	6	1	0	1	8	
	Gupta	7	0	0	0	7	
	Shrivastava	6	1	0	0	7	
	Kurmi	6	0	0	0	6	
	Other mixed castes	79	10	0	0	89	
Socioeconomic status	Lower	2	0	0	0	2	0.204
	Lower middle	102	16	0	1	119	
	Middle lower	1	0	0	0	1	
	Upper	1	0	0	0	1	
	Upper lower	30	0	1	0	31	
	Upper middle	49	4	0	0	53	
Family history of hemoglobinopathy	No	185	20	1	1	207	NA
	Yes	0	0	0	0	0	

The difference in the degree of anemia between different categories of the diagnosis is statistically significant (p=0.017). The most common category of the degree of anemia was mild anemia, consisting of 118 participants, while moderate anemia included 44 participants, and no anemia included 42 participants. The category of severe anemia was rare, including only three participants. For those having β-thalassemia trait, the category of mild anemia is common, while for severe anemia, there are two participants. For HbE trait, the category of mild and moderate anemia is mostly found, while iron deficiency anemia and δβ-thalassemia traits have severe and moderate anemias, correspondingly (Table 4).

**Table 4:** Distribution of anemia severity according to hemoglobinopathy diagnosis HbE: hemoglobin E; β-thalassemia: beta-thalassemia; δβ-thalassemia: delta-beta-thalassemia

Anemia severity	β-Thalassemia trait	HbE trait	Iron deficiency anemia	δβ-Thalassemia trait	Total (n)	P-value (Freeman-Halton exact test)
None	36	6	0	0	42	0.017
Mild	110	8	0	0	118	
Moderate	37	6	0	1	44	
Severe	2	0	1	0	3	
Total	185	20	1	1	207	

The hematological and HPLC parameters showed distinct descriptive patterns across the four diagnostic categories. β-Thalassemia trait had a mean MCV of 69.93±8.75 fL, MCH of 23.82±4.44 pg, and Hb of 9.98±1.25 g/dL, while HbE trait showed slightly higher MCV (71.20±7.11 fL) and MCH (25.03±3.49 pg). HbE trait was characterized by markedly higher HbA₂ (27.57±6.48%) and lower HbA (71.47±6.33%) compared with β-thalassemia trait (HbA₂ 4.76±1.63%; HbA 93.95±1.96%). The iron deficiency anemia and δβ-thalassemia groups showed individual values that were presented descriptively because each group contained only one participant. Therefore, no inferential conclusion should be drawn for these two single-observation groups (Table 5).

**Table 5:** Hematological and HPLC parameters according to diagnostic category Values are presented as mean±SD. "—" indicates that the SD could not be calculated because only one observation was available in that diagnostic category. Iron deficiency anemia and δβ-thalassemia trait groups each comprised one participant and were therefore presented descriptively only and excluded from inferential statistical comparisons. HPLC: high-performance liquid chromatography; MCV: mean corpuscular volume; MCH: mean corpuscular hemoglobin; RDW: red cell distribution width; Hb: hemoglobin; HbA: hemoglobin A; HbA₂: hemoglobin A2; HbF: fetal hemoglobin; HbE: hemoglobin E; SD: standard deviation; ANOVA: analysis of variance; β-thalassemia: beta-thalassemia; δβ-thalassemia: delta-beta-thalassemia

Variable	Category	Mean±SD
MCV (fL)	β-Thalassemia trait	69.93±8.75
	HbE trait	71.20±7.11
	Iron deficiency anemia	68.70±—
	Other variants (δβ-thalassemia)	83.20±—
MCH (pg)	β-Thalassemia trait	23.82±4.44
	HbE trait	25.03±3.49
	Iron deficiency anemia	23.30±—
	Other variants (δβ-thalassemia)	31.00±—
RDW (%)	β-Thalassemia trait	19.29±3.65
	HbE trait	21.23±2.57
	Iron deficiency anemia	32.20±—
	Other variants (δβ-thalassemia)	25.40±—
Hb (g/dL)	β-Thalassemia trait	9.98±1.25
	HbE trait	10.12±1.20
	Iron deficiency anemia	3.10±—
	Other variants (δβ-thalassemia)	8.30±—
HbA (%)	β-Thalassemia trait	93.95±1.96
	HbE trait	71.47±6.33
	Iron deficiency anemia	94.50±—
	Other variants (δβ-thalassemia)	84.60±—
HbA₂ (%)	β-Thalassemia trait	4.76±1.63
	HbE trait	27.57±6.48
	Iron deficiency anemia	3.60±—
	Other variants (δβ-thalassemia)	1.80±—
HbF (%)	β-Thalassemia trait	1.31±0.98
	HbE trait	0.97±0.50
	Iron deficiency anemia	1.90±—
	Other variants (δβ-thalassemia)	13.60±—

The overall clinical and hematological characteristics of the study population are presented in Table 6. The mean maternal age was 26.48±4.76 years. The mean hemoglobin concentration was 9.95±1.33 g/dL, with a mean RBC count of 4.71±1.09 million/mm³. The mean MCV and MCH were 70.11±8.61 fL and 23.97±4.37 pg, respectively. The mean RDW was 19.56±3.71%, while the mean HbA, HbA₂, and HbF levels were 91.74±7.19%, 6.94±7.21%, and 1.33±1.27%, respectively.

**Table 6:** Overall clinical and hematological characteristics of the study population SD: standard deviation; RBC: red blood cell; MCV: mean corpuscular volume; MCH: mean corpuscular hemoglobin; MCHC: mean corpuscular hemoglobin concentration; RDW: red cell distribution width; HbA: hemoglobin A; HbA₂: hemoglobin A2; HbF: fetal hemoglobin

Parameter	Mean±SD
Maternal age (years)	26.48±4.76
Hemoglobin (g/dL)	9.95±1.33
RBC count (million/mm³)	4.71±1.09
MCV (fL)	70.11±8.61
MCH (pg)	23.97±4.37
MCHC (g/dL)	35.18±18.85
RDW (%)	19.56±3.71
HbA (%)	91.74±7.19
HbA₂ (%)	6.94±7.21
HbF (%)	1.33±1.27

## Discussion

This study revealed that 5,285 women of reproductive age attended a tertiary care center in Central Uttar Pradesh, of whom 3.89% were identified as having a hemoglobinopathy. Among the 207 participants who underwent complete diagnostic evaluation, 89.4% were diagnosed with β-thalassemia trait. This study contributes to the epidemiological data for the region, which remains relatively limited compared with screening studies from other parts of the country. Previous evidence has largely been based on studies from other parts of Uttar Pradesh or elsewhere in India, with limited representation of populations from the same geographical and linguistic setting as the present study [1,2].

The prevalence of β-thalassemia trait in India can be compared with the multicenter study by Mohanty et al., who estimated the prevalence of β-thalassemia trait among 56,780 people across six cities in India using standardized methods. They reported an overall prevalence of 2.78%, with state-level variation ranging from 1.48% to 3.64% [2]. The prevalence of β-thalassemia trait in the present study was 3.5%, which is within this reported range. This difference may be partly explained by the present study being conducted in a tertiary care obstetric referral center, where the population may be enriched for anemia and various obstetric complications. Such a setting may increase the likelihood of identifying symptomatic individuals or those referred for further investigation of suspected hemoglobinopathy. Therefore, the higher prevalence observed in this hospital-based study compared with community-based or school-based studies may not necessarily represent the prevalence in the general population of Central Uttar Pradesh.

In a neighboring region of North India, Singh et al. reported a prevalence of β-thalassemia trait of 2.6% among a first-trimester antenatal cohort screened using HPLC at a tertiary care center in Uttarakhand [9]. The slightly higher prevalence observed in the present study may be related to differences in population catchment, referral practices, or the broader clinical spectrum included in the present study, which involved women aged 15-49 years attending both antenatal and outpatient services rather than only women attending first-trimester antenatal clinics.

A complete diagnostic work-up was performed in 207 participants, and β-thalassemia trait was the most common finding (89.4%). This is consistent with its established status as one of the most common hemoglobinopathies reported in northern and central Indian populations [1,2]. However, the diagnostic cohort was enriched with participants who had abnormal or suggestive findings on initial screening; therefore, positive findings were inevitably over-represented compared with the total screened population. Consequently, the distribution of diagnostic categories within the 207 participants should not be directly applied to the entire screened population. Narang et al. similarly reported that β-thalassemia trait was the dominant hemoglobinopathy in a reproductive-age female population attending a tertiary center in Punjab and highlighted the public health importance of identifying carriers in this age group [10].

Among the 207 participants included in the diagnostic analysis, 9.7% had HbE trait. In India, HbE is known to show a geographic gradient, with the highest prevalence reported in the north-eastern states and a decreasing frequency towards the west [2,11]. In their multicenter cohort, Mohanty et al. observed that HbE trait was significantly concentrated in the north-eastern region, with particularly high levels among indigenous populations compared with other ethnic groups [2]. In this context, the presence of HbE trait in almost 10% of the diagnostic cohort from Central Uttar Pradesh is noteworthy, but it should be interpreted cautiously because of the small absolute number of participants with HbE trait (n=20) and the referral-based nature of the diagnostic cohort.

Uttar Pradesh has considerable ethnic and population diversity, and published hospital-based studies from different parts of the state have reported variations in hemoglobinopathy profiles [12]. The design of the present study does not allow us to determine whether the 9.7% proportion of HbE trait in the diagnostic cohort reflects a true increase in the local population or is partly related to selective diagnostic referral. The single occurrence of δβ-thalassemia trait (0.5%) and the single occurrence of iron deficiency anemia without identified hemoglobinopathy (0.5%) should similarly be considered descriptive findings. Meaningful comparisons or inferences cannot be made for these categories because of their very small numbers.

The patterns of hematological and HPLC parameters were generally consistent with the respective diagnostic categories. MCV and MCH did not differ significantly between the groups, which is similar to findings from other Indian screening studies where background nutritional deficiency anemia may reduce the discriminatory value of these indices when used alone [3,7]. Statistically significant differences between the diagnostic groups were observed for RDW, hemoglobin, HbA, HbA₂, and HbF (all p<0.001).

The mean HbA₂ level in the β-thalassemia trait group was 4.76%, which is consistent with the established HPLC diagnostic criterion for heterozygous β-thalassemia, where HbA₂ is typically >3.5% [8]. In the HbE trait group, the markedly elevated HbA₂ fraction (mean: 27.57%) is a recognized HPLC chromatographic feature. On the Bio-Rad D-10 platform, the peak reported as "HbA₂" in HbE samples is predominantly composed of HbE rather than true HbA₂. Therefore, interpretation of this pattern requires consideration of retention time, peak morphology, and the proportion of the remaining HbA fraction, as described in published laboratory protocols [7,8].

The elevated HbF level observed in the δβ-thalassemia trait group (13.6%) was consistent with its corresponding HPLC pattern [8].

The observed association between anemia severity and diagnostic category was statistically significant (p=0.017) and is of clinical interest. Mild anemia was the predominant finding among β-thalassemia trait carriers, which is consistent with the generally mild and stable anemia associated with the heterozygous state. It should be noted, however, that anemia in carrier participants cannot necessarily be attributed to the hemoglobinopathy alone. Nutritional deficiencies, particularly iron deficiency, are common among women of reproductive age and may cause or contribute to anemia in both carriers and non-carriers. Sharma et al. showed that coexisting iron deficiency is common among β-thalassemia heterozygotes, particularly females, and may affect HbA₂ levels in some individuals. This may have implications for the identification of β-thalassemia carriers, particularly in resource-limited settings where serum ferritin or other direct measures of iron status may not be readily available [13]. The present study did not systematically assess iron status in all participants, making it difficult to distinguish the contribution of iron deficiency from that of hemoglobinopathy to the anemia observed among carriers.

No significant associations were found between diagnostic category and consanguinity, religion, caste, or socioeconomic status. However, the small number of participants in the rare diagnostic groups (HbE trait (n=20), δβ-thalassemia trait (n=1), and iron deficiency anemia without hemoglobinopathy (n=1)) limits the statistical power to identify potentially meaningful associations with these social and demographic factors. Therefore, the absence of statistical significance should not be interpreted as evidence that these factors are unrelated to hemoglobinopathy status.

Screening women of reproductive age is strategically important for the identification of hemoglobinopathy carriers because it provides an opportunity for partner testing, genetic counselling, and prenatal diagnosis when both partners are found to carry the same clinically relevant hemoglobinopathy [5]. Supporting evidence from India comes from Agarwal et al., who reported that a program involving HPLC-based carrier identification, partner testing, molecular confirmation, and prenatal diagnosis successfully identified at-risk couples and facilitated the prevention of severely affected births in a multicenter cross-sectional study of prenatal hemoglobinopathy screening [5].

The choice between HPLC and CBC as the initial screening approach has also been evaluated in recent Indian studies. Periyavan et al. reported that an HPLC-first screening strategy identified significantly more hemoglobinopathy carriers than a CBC-first strategy and was slightly more cost-effective. This may be partly because some hemoglobinopathy variants, including certain HbE and HbS carriers, may have near-normal red cell indices and could therefore be missed by a CBC-first strategy [6]. These findings provide support for the use of HPLC in appropriate obstetric tertiary care settings, particularly when laboratory resources and confirmatory testing are available. The findings of the present study also highlight the potential value of improving access to HPLC-based testing in tertiary obstetric healthcare settings in Central Uttar Pradesh.

There are several strengths to this study. The screened population of 5,285 women is substantial for a single-center tertiary care study. Standardized HPLC testing was performed using a validated platform, and hematological parameters were interpreted alongside chromatographic findings to support diagnostic classification. Data were collected prospectively using predefined diagnostic criteria and quality control procedures, including repeat testing of invalid chromatographic runs.

The study also has several limitations. First, the single-center hospital-based design limits the generalizability of the findings, and the present data cannot provide a population-level estimate of hemoglobinopathy prevalence in the wider community. Additionally, no a priori sample size calculation was performed, as all eligible participants screened during the study period were included. Second, only 207 participants from the total screened population of 5,285 had sufficient diagnostic information for the diagnostic analysis. Therefore, the distribution of diagnostic categories among these participants should not be considered representative of the distribution that would be expected in an unselected screened population. Third, molecular confirmation of HPLC-based diagnoses was not performed. Consequently, genotype-level characterization, including identification of specific mutations relevant to prenatal risk assessment, was not available for this cohort. The very small numbers in the δβ-thalassemia trait and no identified hemoglobinopathy (iron deficiency anemia-only) groups (n=1 each) also preclude meaningful statistical comparisons involving these categories. Finally, the lack of systematic assessment of iron status in all participants limits the ability to determine the independent contribution of iron deficiency to anemia among carrier participants. It also cannot be ruled out that concurrent undetected iron deficiency may have influenced HbA₂ levels or contributed to borderline β-thalassemia trait findings in some participants [13].

## Conclusions

Hemoglobinopathies were identified in 3.89% of the women screened at this tertiary care center in Central Uttar Pradesh, with β-thalassemia trait being the most common finding. Anemia severity and several hematological and HPLC parameters differed significantly across the diagnostic groups. These findings highlight the importance of screening women of reproductive age, as identifying carriers can provide an opportunity for partner testing, counselling, and appropriate prenatal evaluation. However, the hospital-based setting and the relatively small diagnostic cohort should be considered when interpreting these findings, as they may not reflect the prevalence in the wider community. Further multicenter studies involving broader community populations and molecular confirmation could provide a clearer picture of the hemoglobinopathy spectrum in the region.
